# Case Report: Metachronous multiple primary neoplasms in a carrier of germline *MSH2* c.958dupA Lynch syndrome

**DOI:** 10.3389/fonc.2026.1958351

**Published:** 2026-10-28

**Authors:** Liqiong Wang, Ping Li, Duanlin Hu, Xiaozhong Wang, Zhiyuan Xu

**Affiliations:** 1 Department of Gynecology, The University of Hong Kong-Shenzhen Hospital (HKU-SZH), Shenzhen, China; 2 Shenzhen Clinical Research Center for Rare Diseases, Shenzhen, China; 3 Department of Pathology, The University of Hong Kong-Shenzhen Hospital, Shenzhen, China; 4 Department of Oncology Medical Center, The University of Hong Kong-Shenzhen Hospital, Shenzhen, Guangdong, China

**Keywords:** EGFR exon 19 deletion, endometrial carcinoma, endoscopic submucosal dissection, gastric high-grade intraepithelial neoplasia, Lynch syndrome, minimally invasive adenocarcinoma, MSH2 gene, multiple primary neoplasms

## Abstract

**Introduction:**

Lynch syndrome is an autosomal dominant hereditary cancer syndrome caused by germline mutations in DNA mismatch repair (MMR) genes, conferring elevated risks of colorectal, endometrial, gastric, ovarian and other malignancies. We report a 38-year-old woman with a germline *MSH2* c.958dupA (p.Thr320Asnfs*13) pathogenic frameshift mutation who developed metachronous multiple primary neoplasms affecting four organ systems over eight years.

**Case description:**

She presented with abnormal uterine bleeding and was diagnosed with endometrioid endometrial adenocarcinoma (FIGO grade 3, stage IA, pT1a N0 M0) at age 38, receiving comprehensive surgical staging followed by vaginal brachytherapy. MMR immunohistochemistry showed loss of MSH2 and MSH6 protein expression with retained MLH1 and PMS2 and positive internal control, confirming dMMR consistent with the germline *MSH2* variant. Eight months later, chest CT detected a right upper lobe lesion; wedge resection confirmed minimally invasive adenocarcinoma (MIA, pT1mi N0 M0, stage IA1). The tumour measured 0.8 cm with negative margins. ARMS-PCR detected an *EGFR* exon 19 deletion (19-DEL); other hotspot mutations were negative. Exploratory markers were assessed but did not guide management. Elastic fibre staining supported the MIA diagnosis. Serial esophagogastroduodenoscopy (2019–2021) documented gastric low-grade intraepithelial neoplasia (LGIN); endoscopic submucosal dissection (2023) achieved curative resection of high-grade gastric intraepithelial neoplasia (HGIN). In April 2026, colonoscopy detected >10 flat micro-polyps (0.2–0.3 cm) in the rectosigmoid colon with NICE type 1 features, treated with argon plasma coagulation without histological sampling; their nature remains undetermined. No recurrence occurred during eight years of follow-up.

**Conclusion:**

This case illustrates the clinical and biological heterogeneity of neoplasms in a germline *MSH2* carrier. The endometrial carcinoma was confirmed dMMR by immunohistochemistry, consistent with the germline *MSH2* variant, and classified as Lynch-associated. The gastric precursor lesion was possibly Lynch-associated. The pulmonary MIA was compatible with a sporadic *EGFR*-driven origin, although a Lynch-associated contribution could not be excluded. Colorectal micro-polyps were of undetermined histological nature. Management should be individualized, and each lesion assessed on its own histology, molecular profile and tissue availability.

## Background

Lynch syndrome is the most prevalent hereditary colorectal cancer syndrome, resulting from germline pathogenic variants in DNA mismatch repair genes *MLH1*, *MSH2*, *MSH6*, and *PMS2* (1). Although colorectal cancer is the hallmark malignancy, endometrial carcinoma is the most common extracolonic tumour among female carriers, with a lifetime risk of 40–60%, and frequently serves as the sentinel tumour (2). Pathogenic *MSH2* variants account for approximately 20% of Lynch syndrome cases and confer substantially increased risks of gastric, ovarian and other extracolonic malignancies (3).

Tumours associated with Lynch syndrome typically manifest at younger ages than sporadic counterparts and display diverse pathological characteristics. A major clinical challenge is the predisposition to synchronous and metachronous multiple primary malignancies across distinct organ systems (4). Gastric cancer is one of the most lethal Lynch-associated malignancies; cumulative lifetime risk reaches 1–13% among *MSH2* mutation carriers, and precursor gastric intraepithelial neoplastic lesions can be captured by regular endoscopic surveillance (5).

We describe a young woman carrying a pathogenic germline *MSH2* variant who developed sequential metachronous lesions affecting the endometrium, lung, stomach and colorectum over eight years. This case emphasises the clinical value of systematic multi-organ surveillance combined with timely intervention and highlights tissue-dependent biological diversity of neoplasms developing under a constitutional predisposition to MMR deficiency. This case report was prepared in accordance with the 2017 CARE guidelines (6). Current guidelines recommend universal mismatch repair screening for newly diagnosed endometrial carcinoma, irrespective of age or family history (7).

## Case description

A 38-year-old woman with premature ovarian insufficiency diagnosed in 2015 and receiving cyclic hormonal replacement therapy presented with intermittent abnormal uterine bleeding from 2016 to early 2018. Physical examination revealed no abdominal guarding, rebound tenderness, or superficial lymphadenopathy; the uterus was mildly enlarged without adnexal tenderness or palpable masses. Pelvic ultrasound in January 2018 identified an intrauterine lesion measuring 13 × 7 mm with ill-defined borders and abundant vascular signals. Serial ultrasonography demonstrated progressive enlargement to 31 × 25 × 21 mm in February and 34 × 23 mm in March 2018.

Diagnostic hysteroscopy on March 14, 2018 revealed a broad-based grey-white exophytic uterine lesion. Partial curettage and complete electrocautery resection of the stalk were performed. Serum CA125 was 35.4 U/mL. Malignancy was suspected, and the patient underwent comprehensive surgical staging on April 18, 2018, including total hysterectomy, bilateral salpingo-oophorectomy, pelvic and para-aortic lymphadenectomy. Intraoperative exploration identified a cauliflower-like exophytic tumour at the uterine fundus measuring approximately 4.0 × 3.3 × 0.7 cm.

Postoperative pathology confirmed endometrioid endometrial adenocarcinoma, FIGO grade 3, stage IA (pT1a N0 M0), with less than half myometrial invasion and focal suspicious lymphovascular invasion based on haematoxylin and eosin staining without confirmatory CD31/D2–40 immunohistochemistry; perineural invasion was absent. A total of 37 lymph nodes were examined: 5 left para-aortic, 3 right para-aortic, 7 left pelvic, and 22 right pelvic lymph nodes. None showed tumour involvement. The cervix, bilateral parametrial soft tissues, bilateral ovaries and fallopian tubes were free of tumour. All surgical margins were tumour-free. Immunohistochemistry demonstrated ER−, PR−, WT1−, with heterogeneous p53 staining consistent with a wild-type pattern.

MMR immunohistochemistry was performed on the endometrial carcinoma specimen using a four-antibody panel (MLH1, PMS2, MSH2, and MSH6). Non-neoplastic cells showed retained expression, confirming adequate internal control staining. The tumour showed loss of MSH2 and MSH6 protein expression, with retained MLH1 and PMS2. This pattern is consistent with the germline *MSH2* c.958dupA variant and confirms that the endometrial carcinoma is dMMR. The endometrial carcinoma is therefore classified as Lynch-associated. Representative histology and MMR immunohistochemistry are shown in **Figure 1**.

**Figure 1:**
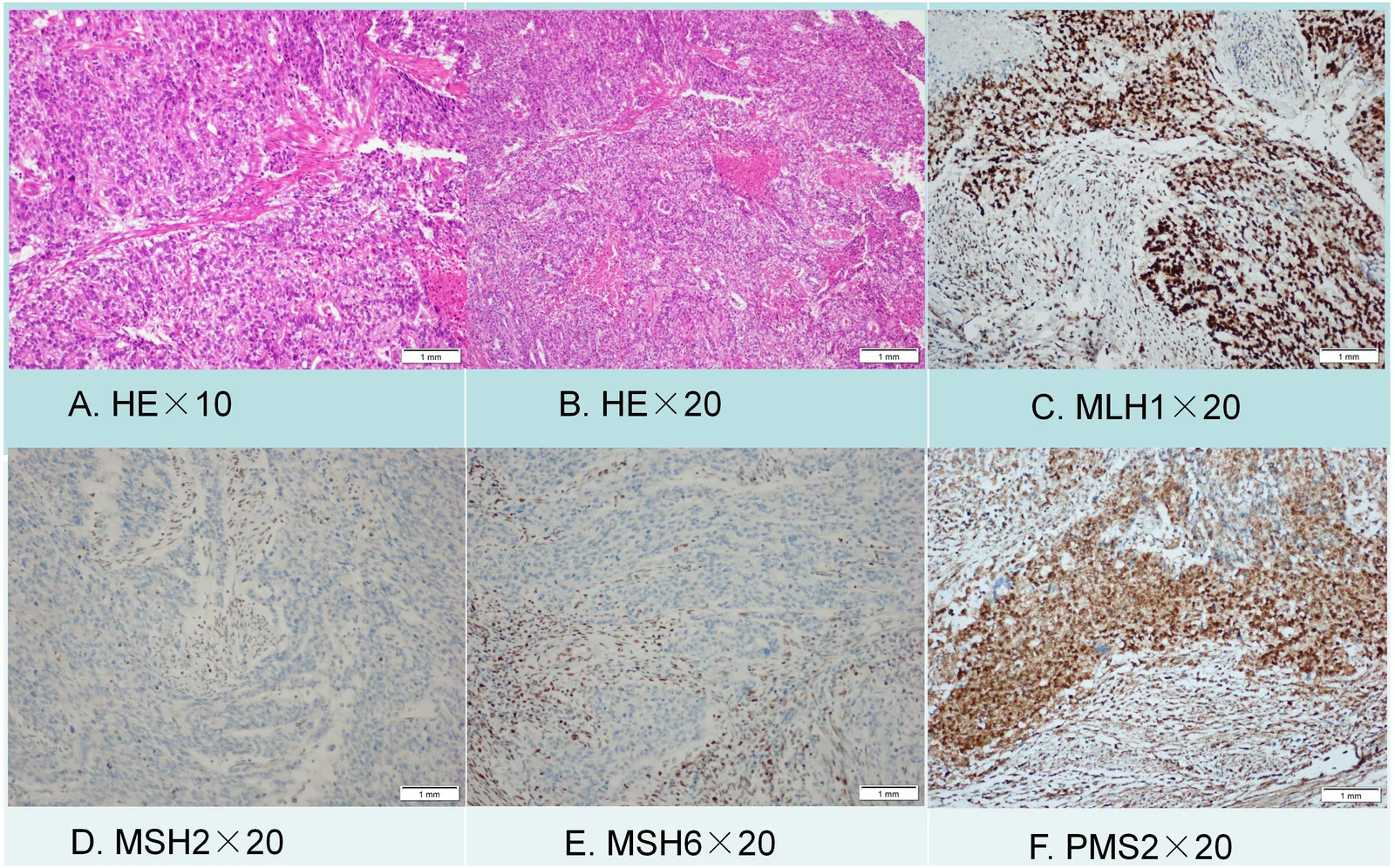
Representative histology and MMR immunohistochemistry of the endometrial carcinoma. **(A)** H&E staining, low magnification (original magnification ×100, 10× objective). **(B)** H&E staining, high magnification (original magnification ×200, 20× objective). **(C)** Retained nuclear MLH1 expression in tumour cells and non-neoplastic cells. **(D)** MSH2 immunohistochemistry showing loss of nuclear expression in tumour cells, with retained expression in non-neoplastic cells as an internal control. **(E)** MSH6 immunohistochemistry showing loss of nuclear expression in tumour cells, with retained expression in non-neoplastic cells as an internal control. **(F)** Retained nuclear PMS2 expression in tumour cells and non-neoplastic cells. Original magnification: ×100 **(A)** and ×200 **(B–F)**. Scale bars: 1 mm.

The patient was treated with adjuvant high-dose-rate vaginal brachytherapy (6 Gy × 5 fractions, total 30 Gy) on an individualized basis. We acknowledge that under the 2021 ESGO/ESTRO/ESP guidelines, unequivocal or substantial LVSI is required to change risk stratification, and suspected LVSI without CD31/D2–40 confirmation is insufficient for definitive assignment to the high-intermediate risk group. No systemic chemotherapy or pelvic external beam radiotherapy was delivered. The patient recovered uneventfully and entered structured oncological surveillance.

Germline genetic testing using a 21-gene hereditary cancer panel on December 11, 2018, identified a heterozygous pathogenic frameshift variant *MSH2* c.958dupA (p.Thr320Asnfs*13) (ClinVar: VCV000091258; dbSNP: rs63749852; transcript NM_000251.3). This finding was independently validated on the same day using a separate 14-gene gastrointestinal cancer panel, confirming the diagnosis of Lynch syndrome. No additional pathogenic variants were detected.

### Diagnostic assessment and diagnostic uncertainty

The level of diagnostic certainty differed among the lesions. The endometrial carcinoma was confirmed histologically and showed loss of MSH2 and MSH6 protein expression with retained MLH1 and PMS2, supporting its classification as a Lynch-associated dMMR tumour. The pulmonary lesion was confirmed as MIA and harboured an *EGFR* exon 19 deletion; however, MMR testing could not be performed because archival tissue was unavailable. The gastric lesion was documented as LGIN on serial biopsies and HGIN in the ESD specimen, but MMR status was not assessed. The colorectal micro-polyps were ablated without histological sampling and therefore remain unclassified. The chronological clinical course and corresponding interventions are presented in **Table 1**. These differences in diagnostic certainty are summarised in **Table 2**.

**Table 1:** Timeline of metachronous neoplastic lesions and therapeutic interventions.

Time	Clinical event	Key findings and interventions
March 14, 2018	Diagnostic hysteroscopy	Endometrioid endometrial adenocarcinoma, FIGO grade 3; ER−, PR−
April 18, 2018	Comprehensive surgical staging	Total hysterectomy + bilateral salpingo-oophorectomy + pelvic and para-aortic lymphadenectomy
April–May 2018	Adjuvant radiotherapy	Vaginal brachytherapy (30 Gy in 5 fractions)
December 11, 2018	Germline genetic testing	Pathogenic frameshift variant *MSH2* c.958dupA (p.Thr320Asnfs*13), identified via 21-gene hereditary cancer panel and independently validated by a 14-gene gastrointestinal cancer panel
December 2018	Surveillance chest CT	Suspicious lesion identified in the right upper pulmonary lobe
January 14, 2019	VATS wedge resection	Minimally invasive adenocarcinoma (pT1mi N0 M0, stage IA1); *EGFR* exon 19 deletion (+); diffuse c-MET (100%, 2+), VEGF (100%, 1+); PD-L1 (−), PD-1 (−)
2019	Surveillance EGD	Gastric low-grade intraepithelial neoplasia (LGIN)
2020–2021	Surveillance EGD	Gastric LGIN
March 2023	Endoscopic submucosal dissection (ESD)	Gastric high-grade intraepithelial neoplasia (HGIN); negative resection margins
April 2026	Surveillance colonoscopy	>10 flat micro-polyps (0.2–0.3 cm, NICE type 1); treated with argon plasma coagulation
2018–2026	Longitudinal oncological surveillance	No recurrence of previously treated tumours during follow-up

BSO, bilateral salpingo-oophorectomy; VATS, video-assisted thoracoscopic surgery; MIA, minimally invasive adenocarcinoma; EGD, esophagogastroduodenoscopy; ESD, endoscopic submucosal dissection; APC, argon plasma coagulation.

**Table 2:** Likelihood of Lynch association for each neoplastic lesion.

Lesion	Year	Histology	MMR/molecular data	Tissue available for MMR?	Likelihood of Lynch association
Endometrial carcinoma	2018	Endometrioid adenocarcinoma, FIGO grade 3, ER−/PR−	MMR IHC: MSH2/MSH6 loss, MLH1/PMS2 retained, internal control positive; p53 wild-type pattern	Yes	Confirmed dMMR; Lynch-associated
Gastric precursor lesion	2019–2023	LGIN on biopsies; HGIN in ESD specimen	Not tested	ESD tissue available; MMR not performed	Possibly Lynch-associated
Pulmonary MIA	2019	MIA, pT1mi N0 M0	*EGFR* exon 19 deletion; exploratory c-MET/VEGF/PD-L1/PD-1 results	No	Compatible with sporadic *EGFR*-driven origin; Lynch association cannot be excluded
Colorectal micro-polyps	2026	Endoscopically identified flat micro-polyps; no histology	Not tested	No	Unknown; adenomatous nature not confirmed
Prior tubulovillous adenoma	2018	Tubulovillous adenoma	Not tested	Unknown	Possible Lynch-associated colorectal neoplasia

### Pulmonary lesion

In December 2018, surveillance chest CT detected a suspicious lesion in the right upper lobe. Video-assisted thoracoscopic wedge resection was completed on January 14, 2019. The tumour measured 0.8 cm in maximum diameter, with no visceral pleural invasion and negative surgical margins.

Histology confirmed minimally invasive adenocarcinoma (MIA, pT1mi N0 M0, stage IA1). Elastic fibre staining (1+) demonstrated focal disruption of alveolar elastic fibres in part of the alveolar septa, providing morphological evidence supporting the diagnosis of MIA by confirming stromal invasion beyond the lepidic growth component.

ARMS-PCR-based molecular testing identified an *EGFR* exon 19 deletion (19-DEL). No mutations were detected in exon 18 (G719X), exon 20 (T790M, S768I, and 20-ins), or exon 21 (L858R and L861Q). Immunohistochemistry revealed diffuse c-MET expression in 100% of tumour cells (intensity 2+), diffuse VEGF expression in 100% of tumour cells (intensity 1+), and negative PD-L1 (22C3) and PD-1 (MRQ-22) staining, with appropriate negative reagent controls to assess non-specific staining. These markers were assessed as exploratory research analyses and did not guide clinical management. Archival lung tissue could not be retrieved for retrospective MMR immunohistochemistry; therefore, the MMR status of the pulmonary tumour remains unknown, and a Lynch-associated origin cannot be definitively excluded. No adjuvant therapy was administered, and no pulmonary recurrence has been observed to date.

### Gastric precursor lesion

Annual surveillance EGD from 2019 to 2021 revealed antral lesions documented as low-grade intraepithelial neoplasia (LGIN) on biopsies obtained in 2019, 2020 and 2021. Endoscopic submucosal dissection was performed in March 2023. Histology confirmed gastric antral high-grade intraepithelial neoplasia (HGIN) with negative resection margins, achieving curative resection.

### Colorectal lesions

Colonoscopy in April 2026 identified more than ten scattered flat micro-polyps (0.2–0.3 cm) in the rectum and sigmoid colon with NICE type 1 morphology. All visible polyps were ablated by argon plasma coagulation without histological sampling. Their adenomatous nature was therefore not confirmed, and they are referred to as endoscopically identified lesions of undetermined histological nature. The patient had undergone polypectomy for a tubulovillous adenoma in 2018.

### Family history

The patient’s mother developed triple-negative breast cancer at approximately 70 years of age. This is reported as family history only and is not interpreted as evidence of an expanded *MSH2* phenotype. Breast cancer associations have been reported for pathogenic variants in other Lynch syndrome genes, including MSH6 and PMS2 (8). No other first- or second-degree relatives had documented colorectal, gastric, endometrial, ovarian or other malignancies.

Over eight years of surveillance (2018–2026), the patient remained asymptomatic. Serial imaging and endoscopies revealed no evidence of recurrence. She maintains disease-free status after curative treatment of endometrial, pulmonary and gastric lesions, with ongoing colorectal surveillance. All procedures were tolerated without adverse or unanticipated events, and adherence to the surveillance programme was documented at each clinical visit.

## Discussion

Several features of this case are relevant to Lynch syndrome management: the multi-organ tumour spectrum, the role of surveillance and timely intervention, and the differing biology of lesions arising in a carrier of a germline MMR defect. The development of metachronous lesions in the endometrium, lung, stomach and colorectum within eight years in one *MSH2* carrier is uncommon. This longitudinal course highlights the importance of guideline-based surveillance across established Lynch syndrome-associated organ systems while illustrating the need for lesion-specific diagnostic assessment.

The endometrial carcinoma occurred at age 38, much younger than the median age of sporadic endometrial cancer, consistent with the early-onset pattern of Lynch-associated malignancy (2). The *MSH2* c.958dupA variant is predicted to cause protein truncation within the ATPase/dimerization domains. Functional data confirming loss of MMR activity for this specific variant are not available. Her history of premature ovarian insufficiency and borderline CA125 elevation is non-specific; neither is a recognized manifestation of Lynch syndrome, and neither changed ovarian cancer surveillance in this patient.

In 2018, universal MMR immunohistochemical screening was not standard practice for endometrial cancer at our institution, so tumour MMR status was not assessed at diagnosis. Germline sequencing was instead initiated because of young age and adverse pathological features. This confirmed Lynch syndrome. MMR immunohistochemistry was subsequently performed on the archived endometrial carcinoma specimen and showed loss of MSH2 and MSH6 protein expression with retained MLH1 and PMS2 and positive internal control, consistent with the germline *MSH2* variant. Upfront tumour MMR immunohistochemistry is now the preferred first-line screening strategy, but germline testing remains a valid diagnostic route when tissue-based screening cannot be completed.

The pulmonary MIA harboured a canonical *EGFR* exon 19 deletion, which is compatible with an independent sporadic driver event. However, a Lynch-associated contribution cannot be excluded because MMR status was not assessed. There is no consistent evidence of increased lung cancer risk in Lynch syndrome. Exploratory immunohistochemical markers did not guide management and are not discussed further. Early detection at stage IA1 allowed curative resection without adjuvant therapy. This single case does not support routine thoracic imaging for all *MSH2* carriers.

Elastic fibre staining helps distinguish AIS from MIA by showing focal disruption of alveolar elastic fibres. It contributes to histological classification and pathological staging. Chest imaging in this patient was part of an individualized surveillance plan, not a standard component of Lynch syndrome surveillance.

Serial EGD showed LGIN on biopsies obtained in 2019–2021, and ESD in 2023 confirmed HGIN with negative margins (9). Under current WHO and Revised Vienna Classification terminology, mild dysplasia and LGIN are synonymous. The previous wording suggesting progression from mild dysplasia to LGIN has been removed. Because forceps biopsies sample only part of a lesion, the change from LGIN on biopsy to HGIN in the ESD specimen may reflect diagnostic upgrading due to sampling limitations rather than true biological progression. This possibility cannot be excluded. Whether gastric LGIN should prompt six-monthly surveillance remains hypothesis-generating and is not supported by current guidelines. Prospective studies are needed before such an approach can be recommended.

Colonoscopy found more than ten flat rectosigmoid micro-polyps (0.2–0.3 cm, NICE type 1) that were treated with APC without biopsy (10). Their adenomatous nature was not confirmed. They are therefore described as endoscopically identified lesions of undetermined histological nature. The decision to ablate without biopsy was made after individualized risk discussion; histological confirmation would have been preferable. The prior tubulovillous adenoma in 2018 is documented separately.

The endometrial tumour had an unusual ER−/PR− immunophenotype. Most sporadic low-grade endometrioid carcinomas express hormone receptors and respond to endocrine therapy; ER/PR negativity is more common in high-grade or serous-type tumours. This profile is notable. MMR immunohistochemistry confirmed loss of MSH2 and MSH6 protein expression, consistent with the germline *MSH2* variant. However, no general conclusion about dMMR-specific biology can be drawn from this single case.

### Endometrial cancer molecular classification

In contemporary endometrial cancer classification, integration of *POLE* mutation status, MMR status and p53 status allows assignment to molecular subgroups with prognostic and therapeutic relevance. In this patient, loss of MSH2/MSH6 protein expression establishes an MMR-deficient phenotype, while p53 immunohistochemistry showed a wild-type pattern. Because *POLE* sequencing was not performed, complete retrospective molecular classification was not possible.

The patient received vaginal brachytherapy alone on an individualized basis. Under the 2021 ESGO/ESTRO/ESP guidelines, unequivocal or substantial LVSI is required to change risk stratification; suspected LVSI without CD31/D2–40 confirmation is not sufficient to assign the patient definitively to the high-intermediate risk group (11). MMR immunohistochemistry confirmed that the endometrial carcinoma is dMMR. The favourable 8-year outcome cannot be extrapolated to MMR-deficient grade 3 endometrial cancers in general and should not be interpreted as evidence supporting treatment de-escalation.

The main observation in this case is the heterogeneity among neoplasms arising in the same *MSH2* carrier. The FIGO grade 3 endometrial carcinoma was confirmed dMMR and remained recurrence-free after treatment. The pulmonary MIA harboured an *EGFR* exon 19 deletion and was successfully treated by limited resection, although its relationship to Lynch syndrome remains uncertain (12). The gastric precursor lesions showed slow endoscopic and pathological changes over years, while the colorectal micro-polyps were of undetermined histological nature. Taken together, these findings illustrate the heterogeneity of neoplasms occurring in a single Lynch syndrome carrier. They also support assessing each lesion according to its own histology, stage and available molecular findings rather than assuming a common pathogenesis.

### Molecular assessment of individual lesions

This case underscores that a germline *MSH2* pathogenic variant establishes cancer predisposition but does not establish the molecular origin of every subsequent lesion. Ideally, assessment of a new tumour in a Lynch syndrome carrier begins with tumour MMR/MSI status, followed, when clinically relevant and tissue is available, by evaluation of somatic MMR alterations and tumour-specific driver mutations. More extensive sequencing may be useful in selected cases in which the relationship to Lynch syndrome remains uncertain.

In this patient, the endometrial carcinoma showed loss of MSH2 and MSH6 protein expression and can be confidently considered Lynch-associated. The evidence for the other lesions is less complete. The pulmonary adenocarcinoma carried an *EGFR* exon 19 deletion but was not tested for MMR deficiency, the gastric lesion was not molecularly characterized, and no tissue was obtained from the colorectal micro-polyps. Their relationship to the germline *MSH2* variant therefore remains uncertain to varying degrees. A conceptual overview of germline predisposition and lesion-specific findings in this patient is shown in **Figure 2**.

**Figure 2:**
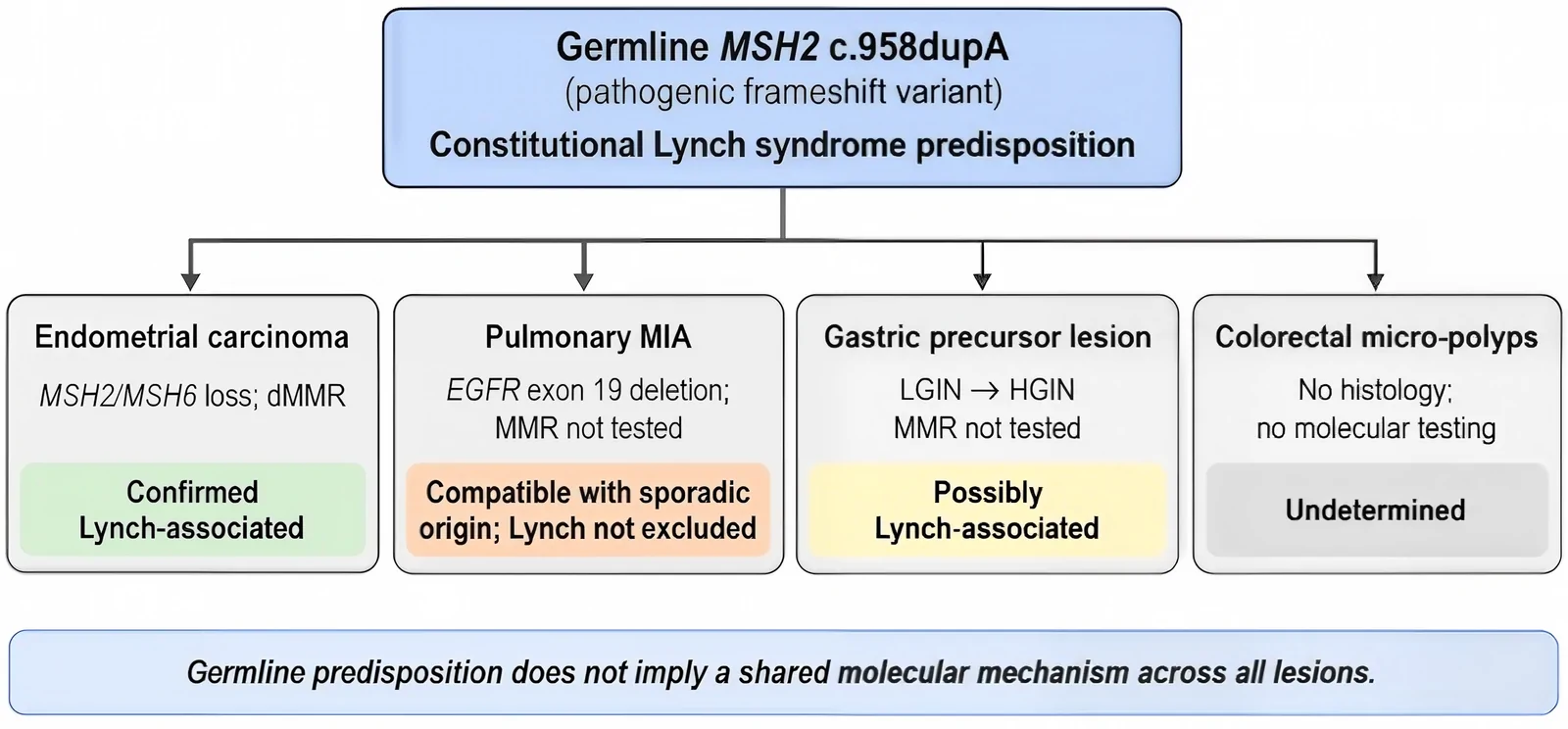
Germline predisposition and lesion-specific findings in a germline *MSH2* carrier. The germline *MSH2* c.958dupA variant establishes Lynch syndrome predisposition, whereas the evidence linking individual lesions to MMR deficiency differs according to the available pathological and molecular data. MMR deficiency was confirmed only in the endometrial carcinoma.

Current NCCN, SGO and ESGO guidance supports universal screening for Lynch syndrome in all patients newly diagnosed with endometrial carcinoma, irrespective of age or family history (7). This patient had no family history of classic Lynch-associated tumours at presentation, confirming that family history alone is insufficient to identify carriers. After diagnosis, established Lynch syndrome surveillance should follow current organ-specific guidelines and individual risk assessment. Additional investigations outside standard Lynch syndrome surveillance should be considered according to individual clinical indications rather than inferred from this single case.

## Strengths and limitations

This single case report cannot provide definitive or generalisable conclusions. Its principal strengths are the 8-year longitudinal follow-up, the sequential documentation of neoplastic lesions across multiple organ systems, and the availability of lesion-specific pathological and selected molecular findings. In particular, the endometrial carcinoma was confirmed to be MMR-deficient, with loss of MSH2 and MSH6 protein expression consistent with the germline *MSH2* pathogenic variant.

Molecular characterization was incomplete, largely because several specimens were obtained at outside institutions or were no longer available. MMR status was established only for the endometrial carcinoma, while the pulmonary tumour underwent targeted *EGFR* testing without MMR assessment. MMR testing was not performed for the gastric lesion, and no tissue was obtained from the colorectal micro-polyps. Tumour sequencing, MSI analysis and assessment of a somatic *MSH2* second hit were therefore unavailable. As a result, only the endometrial carcinoma can be confidently linked to Lynch syndrome on the available tumour-based evidence.

Additional limitations include the lack of CD31/D2–40 confirmation of suspected LVSI, the possibility that the change from gastric LGIN on biopsy to HGIN in the ESD specimen reflects sampling differences rather than biological progression, and the absence of functional studies specifically assessing the *MSH2* c.958dupA variant. These limitations emphasize that the present case should be interpreted as hypothesis-generating rather than as evidence of causality or a basis for changing surveillance guidelines.

### Patient perspective

The patient was fully cooperative and consented to the publication of this case report. Because part of her care had been delivered at other institutions, she collected and provided all relevant outside medical records, including pathology and endoscopy reports, to facilitate the preparation of this report. She had been under the care of the same clinical team throughout follow-up and expressed a strong willingness to share her experience. She considered it an honour that her case might help other patients with Lynch syndrome and hoped it would improve clinical awareness of this condition. She also expressed deep appreciation for the care she received, stating that she felt her life had been saved. She mentioned that she had previously been very anxious about gynecological examinations, but her experience with the clinical team reduced that fear. She found the multi-organ surveillance schedule acceptable and tolerated the procedures well, and she emphasised the importance of regular follow-up and clear communication with her clinical team.

## Conclusions

Current NCCN, SGO and ESGO guidance supports universal screening for Lynch syndrome in all patients newly diagnosed with endometrial carcinoma, irrespective of age or family history. We report a young woman with germline *MSH2* c.958dupA who developed multiple metachronous lesions over eight years. The endometrial carcinoma was confirmed to be dMMR by immunohistochemistry, with loss of MSH2 and MSH6 protein expression, consistent with the germline *MSH2* variant, and is classified as Lynch-associated. The gastric precursor lesion was possibly Lynch-associated. The pulmonary MIA was compatible with a sporadic *EGFR*-driven origin, although a Lynch-associated contribution could not be excluded. The colorectal micro-polyps were of undetermined histological nature. Management should be individualized and based on the stage, histology and molecular profile of each lesion. Prospective studies are needed to better define organ-specific surveillance strategies and the clinical role of lesion-specific molecular characterization in Lynch syndrome.

## Data Availability

The original contributions presented in the study are included in the article/supplementary material. Further inquiries can be directed to the corresponding author.
